# Ecto-Nucleotidase Activities of Promastigotes from *Leishmania (Viannia) braziliensis* Relates to Parasite Infectivity and Disease Clinical Outcome

**DOI:** 10.1371/journal.pntd.0001850

**Published:** 2012-10-11

**Authors:** Pauline M. Leite, Rodrigo S. Gomes, Amanda B. Figueiredo, Tiago D. Serafim, Wagner L. Tafuri, Carolina C. de Souza, Sandra A. L. Moura, Juliana L. R. Fietto, Maria N. Melo, Fátima Ribeiro-Dias, Milton A. P. Oliveira, Ana Rabello, Luís C. C. Afonso

**Affiliations:** 1 Laboratório de Imunoparasitologia, DECBI/NUPEB, Universidade Federal de Ouro Preto, Minas Gerais, Brazil; 2 Departamento de Patologia Geral, ICB, UFMG, Belo Horizonte, Minas Gerais, Brazil; 3 Departamento de Bioquímica e Biologia Molecular, Universidade Federal de Viçosa, Minas Gerais, Brazil; 4 Universidade Federal de Minas Gerais, Belo Horizonte, Brazil; 5 Instituto de Patologia Tropical e Saúde Publica, Universidade Federal de Goiás, Goiânia, Goiás, Brazil; 6 Centro de Pesquisas René Rachou- FIOCRUZ, Belo Horizonte, Minas Gerais, Brazil; Yale School of Public Health, United States of America

## Abstract

**Background:**

*Leishmania (Viannia) braziliensis* has been associated with a broad range of clinical manifestations ranging from a simple cutaneous ulcer to destructive mucosal lesions. Factors leading to this diversity of clinical presentations are not clear, but parasite factors have lately been recognized as important in determining disease progression. Given the fact that the activity of ecto-nucleotidases correlates with parasitism and the development of infection, we evaluated the activity of these enzymes in promastigotes from 23 *L. braziliensis* isolates as a possible parasite-related factor that could influence the clinical outcome of the disease.

**Methodology/Principal Findings:**

Our results show that the isolates differ in their ability to hydrolyze adenine nucleotides. Furthermore, we observed a positive correlation between the time for peak of lesion development in C57BL/6J mice and enzymatic activity and clinical manifestation of the isolate. In addition, we found that *L. (V.) braziliensis* isolates obtained from mucosal lesions hydrolyze higher amounts of adenine nucleotides than isolates obtained from skin lesions. One isolate with high (PPS6m) and another with low (SSF) ecto-nucleotidase activity were chosen for further studies. Mice inoculated with PPS6m show delayed lesion development and present larger parasite loads than animals inoculated with the SSF isolate. In addition, PPS6m modulates the host immune response by inhibiting dendritic cell activation and NO production by activated J774 macrophages. Finally, we observed that the amastigote forms from PPS6m and SSF isolates present low enzymatic activity that does not interfere with NO production and parasite survival in macrophages.

**Conclusions/Significance:**

Our data suggest that ecto-nucleotidases present on the promastigote forms of the parasite may interfere with the establishment of the immune response with consequent impaired ability to control parasite dissemination and this may be an important factor in determining the clinical outcome of leishmaniasis.

## Author Summary

Cutaneous leishmaniasis is a widespread tropical disease caused by different species of *Leishmania* protozoa that are transmitted by infected sandflies. Clinical presentations are extremely diverse and dependent on a variety of parasite and host factors that are poorly understood. *Leishmania (V.) braziliensis* infection may result in a devastating disease manifestation characterized by the development of destructive lesions in the oral, nasal, and pharyngeal mucosal. Ecto-nucleotidases are enzymes that are involved in the hydrolysis of extracellular nucleotides. These enzymes have been shown to correlate with virulence of *Leishmania* parasites. In this work, we evaluated the ecto-nucleotidase activity of promastigotes from the twenty three different *L. braziliensis* isolates. We demonstrated that isolates obtained from mucosal lesions present higher levels of ecto-nucleotidase activity than those from cutaneous lesions. In addition, we show that in the murine model of cutaneous leishmaniasis, promastigote forms of parasite with higher activity induce a delayed/decreased immune response that may correlate with spreading of the parasites throughout the body. Thus, we propose that the level of ecto-nucleotidase activity of promastigotes may be a marker for the development of severe clinical forms of cutaneous leishmaniasis and also a possible target for future therapeutic intervention.

## Introduction

*Leishmania* is the etiological agent of leishmaniasis, a parasitic disease with diverse clinical manifestations in human beings and other mammals. The parasite presents two main stages in their life cycle: the flagellated mobile promastigote, which multiply in the midgut of the sandfly vector and non-motile amastigotes, obligate intracellular forms that live inside host macrophages. This cell differentiation involves numerous changes and is crucial for *Leishmania* pathogenicity [1], [2]. *Leishmania braziliensis* is the species responsible for the majority of cases of human cutaneous leishmaniasis (CL) in Brazil; usually it causes single self-limited cutaneous ulcers at the site of parasite delivery; however, parasites may also metastasize and produce mucosal lesions, usually in mouth, nose, pharynges, and larynges. The mucosal involvement may occur simultaneously to the cutaneous disease (mucocutaneous form) or months to years after the spontaneous or treatment-induced healing of the cutaneous lesion (mucosal form). In both situations mucosal involvement is serious but the latter more often leads to destructive mucosal involvement with disfiguring scars [3]. In humans, resistance to infection by *L. (V.) braziliensis* is associated with the early establishment of a type 1 immune response along with the control of exacerbated inflammatory responses [4]–[6]. In murine models of infection, interferon gamma (IFN-γ) has been shown to act in synergy with another macrophage derived cytokine, tumor necrosis factor alpha (TNF-α), in activating macrophages to synthesize nitric oxide (NO), a potent microbicidal agent that leads to killing of intracellular parasites [7], [8].

Factors or mechanisms leading to the diversity of clinical presentations are not well known. Although the involvement of host immune response [4], [5], [9]–[12], and, a limited number of parasite factors also have lately been recognized as important [13], [14].

Several virulence factors have been associated with the establishment of *Leishmania* infection, including lipophosphoglycan (LPG), gp63 and other proteases. These factors are involved in the establishment of intracellular parasitism as well as in the inhibition of host immune response [15], [16]. Components of extracellular ATP metabolism pathway are emerging candidates to determine the virulence of these parasites, since ATP and adenosine (Ado), a product of AMP hydrolysis, are able to influence the immunological response of the host and, in consequence, the parasite establishment [17]–[21].

Due to their incapacity to *de novo* synthesize purine nucleotides, *Leishmania* parasites need to obtain extracellular nucleosides to feed the salvation pathway for purine nucleotides synthesis. This is achieved through the action of extracellular enzymes [22], amongst them, the ecto-nucleoside triphosphate dyphosphohydrolase (E-NTPDase or apyrase) which will hydrolyze ATP to ADP and then to AMP and the 5′-nucleoside monophosphate phosphohydrolase (5′-nucleotidase or 5′-NT), which produces Ado by removing the phosphate group from AMP. Ado is, then internalized via specific transporters [23].

An increase in the levels of extracellular ATP is interpreted by the immune system as a danger signal and triggers an inflammatory response [24]. On the other hand, extracellular Ado, acting on P1 receptors, modulates the inflammatory response by increasing the intracellular cAMP concentration [25].

Previous studies from our group corroborate the hypothesis that enzymes involved in the extracellular metabolism of nucleotides can also act as virulence factors for parasites. In a comparative study with metacyclic promastigotes of *Leishmania (Leishmania) amazonensis*, *Leishmania (Leishmania) major* and *L. (V.) braziliensis*, a potential correlation between enzymatic activity and virulence *in vivo* was demonstrated [18]. More recently, we demonstrated that long-term culture of *L. (L.) amazonensis* promastigotes results in a decreased ability to hydrolyze nucleotides that is associated with loss of virulence [17].

Since ecto-nucleotidases have a crucial role in metabolism of extracellular nucleotides, which can be correlated to parasitism and the development of infection, we focused our study on the activity of these enzymes in *L. (V.) braziliensis* isolates from patients as a possible parasite-related factor that could influence the clinical presentation of disease. Moreover, we also examined whether ecto-nucleotidases can be correlated with the control of immune response in the infection of C57BL/6J mice and J774-macrophages, as well as the infection and activation of dendritic cells (DC). Our results show that parasites with high ecto-nucleotidase activity are able to modulate the host immune response by inhibiting macrophage microbicidal mechanisms and DC activation. In addition, we observed that promastigotes from the *L. (V.) braziliensis* isolates obtained from mucosal lesions hydrolyze higher amounts of adenine nucleotides than isolates obtained from cutaneous lesions, indicating that differences in the enzymatic activity may influence disease outcome in patients with *L. (V.) braziliensis* infection.

## Methods

### Animals and Parasites

Female C57BL/6J mice (4–8 weeks old) were obtained from the University's animal facility. Animals were given water and food *ad libitum*. The *L. (Viannia) braziliensis* parasites were obtained from the Oswaldo Cruz Institute *Leishmania* collection (Coleção de *Leishmania* do Instituto Oswaldo Cruz, CLIOC), Leishmaniasis Immunobiologic Bank (Leishbank) of the Brazilian Mid-West region in the Tropical Pathology and Public Health Institute, Federal University of Goias (UFG) and Centro de Referências em Leishmanioses do Centro de Pesquisas René Rachou-Fiocruz (CRL-CPqRR) (Table 1). Clinical forms used in the study were those defined by the cell bank from where the isolates were obtained. Parasites were cultured in Grace's insect medium (Sigma-Aldrich, St. Louis, MO, USA) supplemented with 10% heat-inactivated fetal calf serum (FCS – LGC, Cotia, SP, Brazil), 2 mM l-glutamine (Gibco BRL, Grand Island, NY, USA) and 100 U/mL penicillin G potassium (USB Corporation, Cleveland, OH, USA), pH 6.5, at 25°C. Parasites were kept in culture for no more than twenty passages. Metacyclic promastigotes were purified by gradient centrifugation of parasites at the stationary phase of culture (day 5) over Ficoll 400 (Sigma-Aldrich), as previously described [18]. The amastigote forms were obtained from J774 infected macrophages as previously described [26] except for the modification of the incubation temperature of the suspension containing the macrophages and the parasites (33°C).

**Table 1:** Strains of *Leishmania* used in this study.

Isolates^a^	Clinical form	Geographical origin	Identification by
IOC -L 2463, MHOM/BR/2001/JOLIVAL	Mucocutaneous	Bahia	CLIOC^b^
IOC -L 2468, MHOM/BR/2001/LTCP14183	Mucocutaneous	Bahia	CLIOC
IOC -L 2480, MHOM/BR/2001/LTCP13980	Mucocutaneous	Bahia	CLIOC
IOC -L 2481, MHOM/BR/2000/LTCP13490	Cutaneous	Bahia	CLIOC
MHOM/BR/2009/ASL (ASL9m)	Mucosal	Goiás	IPTSP/UFG^c^
MHOM/BR/2009/ILM (ILM9m)	Mucosal	Goiás	IPTSP/UFG
MHOM/BR/2008/JBC (JBC8m)	Mucosal	Goiás	IPTSP/UFG
MHOM/BR/2006/PPS (PPS6m)	Mucosal	Bahia	IPTSP/UFG
MHOM/BR/2006/EFSF (EFSF6)	Cutaneous	Goiás	IPTSP/UFG
MHOM/BR/2010/GVN (GVN10)	Cutaneous	Amapá	IPTSP/UFG
MHOM/BR/2006/HPV (HPV6)	Cutaneous	Tocantins	IPTSP/UFG
MHOM/BR/2003/IMG (IMG3)	Cutaneous	Goiás	IPTSP/UFG
MHOM/BR/2005/RPL (RPL5)	Cutaneous	Pará	IPTSP/UFG
AMAC	Cutaneous	Minas Gerais	CRL - CPqRR^d^
CHP	Cutaneous	Minas Gerais	CRL - CPqRR
ET	Cutaneous	Minas Gerais	CRL - CPqRR
JRG	Cutaneous	Minas Gerais	CRL - CPqRR
LCA	Cutaneous	Minas Gerais	CRL - CPqRR
NSL	Cutaneous	Minas Gerais	CRL - CPqRR
RS	Cutaneous	Minas Gerais	CRL - CPqRR
SAP	Cutaneous	Minas Gerais	CRL - CPqRR
SSF	Cutaneous	Minas Gerais	CRL - CPqRR
WLC	Cutaneous	Minas Gerais	CRL - CPqRR

a *L. (Viannia)* braziliensis.

b CLIOC: *Leishmania* Type Culture Collection, Instituto Oswaldo Cruz.

c IPTSP/UFG: Leishmaniasis Immunobiologic Bank (Leishbank) of the Brazilian Mid-West region in the Tropical Pathology and Public Health Institute, Federal University of Goias (UFG).

d CRL – CPqRR: Centro de Pesquisas René Rachou-Fiocruz.

### Ethics Statement

The protocols to which animals were submitted were approved by the Universidade Federal de Ouro Preto Ethical Committee on Animal Experimentation (OFíCIO CEP N°. 005/2009) and followed the guidelines from the Canadian Council on Animal Care.

All isolates were obtained from pre-established collections from anonymized samples. IRB approval is not required for the use of the parasite isolates and hence not sought.

### Enzymatic activity measurement

ATPase, ADPase and 5′-nucleotidase activities were measured by incubation of intact parasites for 1 hr at 30°C in a mixture containing 116 mM NaCl, 5.4 mM KCl, 5.5 mM D-glucose, 5 mM MgCl_2_, and 50 mM Hepes–Tris buffer, in the presence of ATP, ADP or AMP (Sigma-Aldrich) 5 mM [27]. The reaction was started by the addition of living stationary phase promastigotes or amastigotes isolated from infected J774 macrophages and terminated by the addition of ice cold HCl 0.2 M [28]. Nonspecific hydrolysis was determined by adding the parasites after the reaction was stopped. Parasite suspensions were pelleted and aliquots of supernatant were used for the measurement of released inorganic phosphate (Pi) as previously described [29]. Enzymatic activities were expressed as nmol of Pi released by 1.0×10^8^ parasites in 1 hr.

### Infection

C57BL/6J mice were inoculated in the left hind footpad with 1.0×10^7^ stationary phase promastigotes and lesion development was followed weekly with a dial micrometer (Serie 7; NO. 7301; Mitutoyo). The results were expressed as the difference between measures of infected and contra lateral non-infected footpad [30].

### Parasite load estimation

The number of parasites in the footpad was estimated by a limiting dilution assay [30]. Mice were sacrificed and the whole lesion was removed and ground in Grace's insect medium, pH 6.5, in a glass tissue grinder. Tissue debris was removed by centrifugation at 50× *g* at 4°C/1 min, and supernatant was transferred to another tube and centrifuged at 1540× *g* at 4°C/15 min. The pellet was resuspended in 0.5 mL Grace's insect medium supplemented with 10% heat-inactivated FCS, 2 mM l-glutamine and 100 U/mL penicillin G potassium, pH 6.5. Parasite suspension was, then, serially diluted in duplicates in a final volume of 200 µL in 96-well plates. Pipette tips were replaced for each dilution. Plates were incubated for 15 days at 25°C and examined under an inverted microscope for the presence of parasites. Results were expressed as −log of the parasite titer corresponding to the last dilution in which parasites were detected.

### Histology and immunohistochemistry

Footpad lesions from C57BL/6J mice were harvested, embedded in paraffin and 4 µm-thick sections stained with hematoxylin and eosin (HE) and examined under a light microscope. Deparaffined slides were hydrated and incubated with 4% hydrogen peroxide (30vv) in 0.01 M Phosphate Buffered Saline (PBS; pH 7.2) to block endogenous peroxidase activity, followed by incubation with normal goat serum (1∶100 dilution) to block non-specific immunoglobulin absorption. Heterologous hyperimmune serum from rabbit inoculated with *Leishmania infantum* extract [31] was diluted 1∶800 with BSA 0.1% and employed as the primary antibody. Slides were incubated in a humid chamber at 4°C for 18–22 h, washed with PBS, incubated with biotinylated goat anti-mouse and anti-rabbit Ig (Dako, Carpinteria, CA, 192 USA; LSAB2 kit), washed in PBS, and incubated with streptavidin-peroxidase complex (Dako; LSAB2 kit) for 20 min at room temperature. Slides were treated with 0.024% diaminobenzidine (Sigma-Aldrich) and 0.16% hydrogen peroxidase (30vv), dehydrated, cleared, counterstained with Harris's hematoxylin and mounted with cover slips [31]. The images were captured in a Leica DM5000B microscope with a coupled camera DFC300FX using the program Leica Application Suite (version 2.4.0 R1, Leica Microsystems Ltd., Heerbrugg, Switzerland).

### Infection of J774 cells

J774 cells were plated at 1×10^6^ cells/mL onto round coverslips in Dulbecco's minimal essential medium (Sigma-Aldrich) containing 10% FCS, 2 mM l-glutamine, 100 U/mL penicillin G potassium, 25 mM N-2-hydroxiethylpiperazine-N′-2-ethanosulfonic acid (HEPES; Sigma-Aldrich) and 50 µM β-mercaptoetanol (Pharmacia Biotech AB, Uppsala, Sweden) in 24-well plates. Cells were incubated for 90 min at 37°C, 5% CO_2_. Non-adherent cells were removed by washing with warm phosphate-buffered saline (PBS). Promastigotes or amastigotes isolated from infected J774 macrophages were added to the culture at a 5∶1 parasite to cell ratio. After 3 hr co-culture, cells were washed with PBS to remove non-internalized parasites and coverslips were collected to evaluate infectivity. Fresh medium was added to the cultures and the macrophages were stimulated with 10 U/mL IFN-γ and 100 pg/mL lipopolysaccharide (LPS). After 72 hr, coverslips were collected for evaluation of infectivity and supernatants were collected for measurement of nitrite and interleukin-10 (IL-10) production. Coverslips were fixed in methanol for 10 min (Vetec Fine Chemistry), dried and stained using the kit Panótico Rápido (Laborclin, Pinhais, PR, Brazil), according to manufacturer's instructions. The analysis was performed using an Olympus BX50 optical microscope (Olympus, Center Valley, PA, USA). The number of infected and uninfected cells and the number of parasites present in infected cells were determined. A minimum of 200 macrophages per coverslip was examined.

Quantification of NO produced by the cells was performed by the indirect Griess method to detect nitrite [32], and the production of IL-10 levels was evaluated by indirect enzyme-linked immunosorbent assay (ELISA) (PeproTech Inc., Rock Hill, NJ, USA), according to manufacturer's specifications, in 72 hr supernatants.

### DC infection and flow cytometry

Bone-marrow derived dendritic cell (BMDC) were obtained from C57BL/6J bone marrow as previously described [33]. Briefly, bone marrow cells were isolated from the femur and tibia of C57BL/6J mice. The suspension was centrifuged and cells cultured in RPMI-1640 (Sigma-Aldrich) supplemented with 10% FCS, 2 mM l-glutamine, 100 U/mL penicillin G potassium, and 50 µM β-mercaptoetanol, pH 7.2. Cells were plated in Petri dishes at a concentration of 3×10^5^ cells/mL, and incubated at 37°C/5% CO_2_. Granulocyte-Macrophage Colony-Stimulating Factor (GM-CSF) (R&D Systems, Minneapolis, MN, USA) was added to each plate on the days 0, 3 and 6, at the concentration of 3 ng/mL (1050 U/mL). Non adherent DC were collected on the 9^th^ day of culture. 5(6)-carboxyfluorescein diacetate *N*-succinimidyl ester (CFSE)-labeled metacyclic promastigotes and BMDCs were co-incubated (1∶3 cell to parasite ratio) at 33°C/5% CO_2_/3 hr. To ensure full activation of these cells, LPS (Sigma-Aldrich) at a concentration of 2 µg/mL was then added to the culture and the cells were incubated at 37°C/5% CO_2_ for up to 17 hr. Infected BMDCs were submitted to analysis by flow cytometry. For flow cytometry analysis, cells at a concentration of 1×10^7^ cells/mL in PBS/1% bovine serum albumin (BSA) were submitted to FcγR blocking in the presence of anti-mouse CD16/CD32 (eBioscience, San Diego, CA, EUA). Twenty-five µL of the cell suspension were incubated with a combination of desired antibodies at 4°C/30 min, protected from light. The antibodies used were: anti-mouse CD11c (HL3 clone – BD Pharmingen, San Diego, CA, EUA), anti-mouse MHCII (M5 114.15.2 clone), anti-mouse CD86 (GL1 clone – eBioscience), and their respective isotype controls. The suspension was centrifuged and the cells were washed in PBS, pH 7.2 and resuspended in a solution of 1% paraformaldehyde, 47.7 mM sodium cacodylate, and 113 mM NaCl, pH 7.2. The samples were analyzed in BD FACSCalibur™ flow cytometer. Cell acquisition was performed using BD CellQuest™ Pro software. Data analysis was performed using FlowJo software (Tree Star, Ashland, OR, USA).

### Statistical analysis

Student's *t*-test, ANOVA analysis with Bonferroni post-test and Spearman's test were performed using Prism 5.0 software (GraphPad Software, La Jolla, CA, USA). p<0.05 was considered statistically significant.

## Results and Discussion

Infection by *L. (V.) braziliensis* can cause distinct clinical manifestations, amongst which the development of often mutilating mucosal lesions that may cause permanent impairments to the digestive and respiratory tracts. The reasons for the development of the different clinical manifestations are poorly understood and may involve the host immune response as well as parasite associated virulence factors.

Although mucosal leishmaniasis is the most devastating consequence of *L. (V.) braziliensis* infection in humans, consistent correlation between the source of the clinical isolate and the size of lesions developed in mice infected with different isolates of *L. (V.) braziliensis* obtained from cutaneous or mucosal/mucocutaneous lesions has yet not been evaluated.

**Figure 1:**
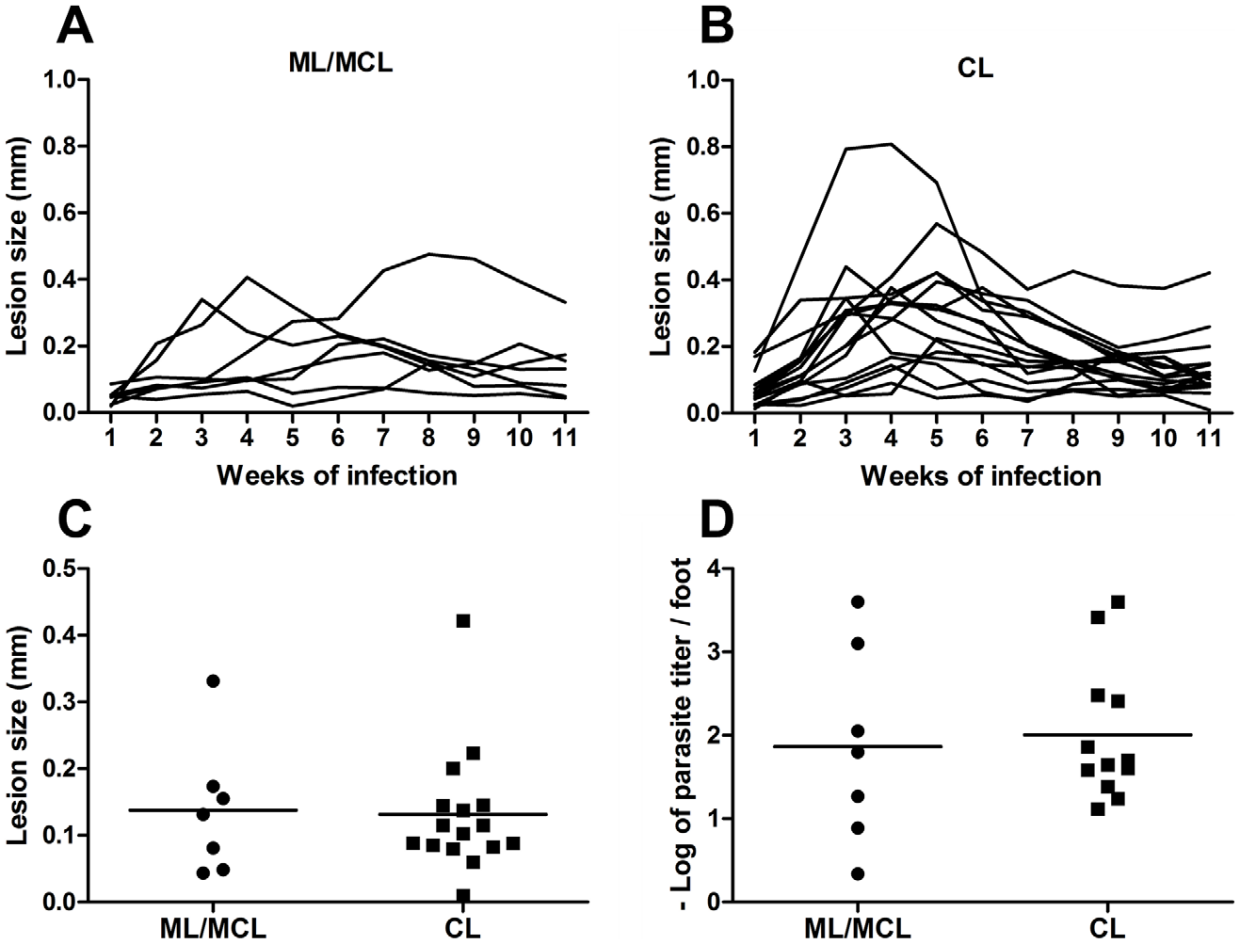
Course of infection of *Leishmania (V.) braziliensis* isolates in C57BL/6J mice. C57BL/6J mice were inoculated in the footpad of the left hind leg with 10^7^ promastigotes obtained from mucosal (ML)/mucocutaneous (MCL) lesions (A) or cutaneous lesions (CL) (B). Lesion sizes were measured weekly. The lesion size was defined as the difference between the infected and uninfected contralateral footpad from two independent experiments with four mice per group with the exception of isolates RS, SAP, IMG3 and RPL5 (one experiment each). Each line represents a distinct isolate and was drawn based on the mean lesion size for each time point. Error bars were not included to facilitate visualization. Eleven weeks after infection, animals were sacrificed and the relationship between lesion size and clinical form (C) as well the parasite load by limiting dilution technique was determined (D). Each point represents a different isolate. The line represents the mean of the group. Statistical analysis was performed by Students's *t*-test.

We decided to compare the course of infection in C57BL/6J mice of isolates obtained from mucosal/mucocutaneous lesions to those from cutaneous lesions. Thus, C57BL/6J mice were inoculated with each of the 23 isolates of *L. (V.) braziliensis* (Table 1) and lesion development monitored weekly. As shown in Figure 1 (A and B) no consistent differences in the lesion size after 11 weeks of infection were observed between the two groups of isolates. With the exception of a few isolates from each group, which developed small but permanent lesions, mice inoculated with isolates from either group were, in general, able to control infection and resolve the lesion. Comparison of the lesion size after eleven weeks of infection showed no statistical difference between the two groups (Figure 1C). In addition, parasitism at the site of infection was similar in both groups (Figure 1D). Thus, our data demonstrated that the ability to develop severe mucosal lesions in patients does not correlate with the development of larger lesions in the murine model.

Several hypotheses have been proposed for the function of ecto-ATPases in trypanosomatids, which include acquisition of Ado from the media, necessary for normal growth, modulation of parasite infection and virulence, and involvement in cellular adhesion [17], [27], [34]–[43].

**Figure 2:**
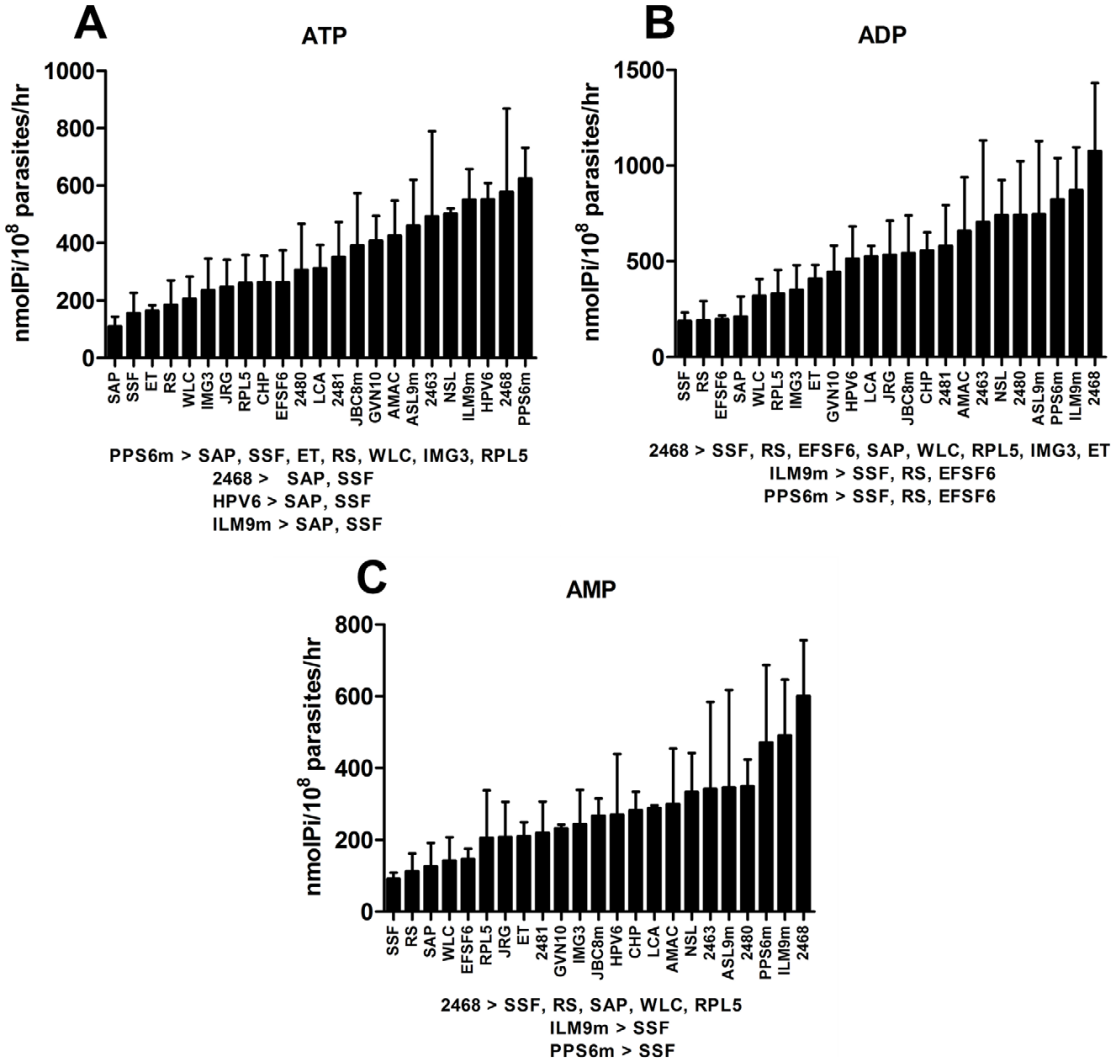
*L. (V.) braziliensis* isolates differ in their ability to hydrolyze adenine nucleotides. Promastigotes were isolated on the 5th day of culture and incubated with ATP (A), ADP (B) and AMP (C) for 1 hr at 30°C. Enzymatic activity was evaluated by the measurement of inorganic phosphate released. Bars represent the mean+standard deviation (SD) of three or more independent experiments performed in triplicates. Significant differences are shown below each graph. Statistical analysis was performed by one-way ANOVA followed by Bonferroni post-test.

It has been suggested that the ecto-nucleotidase activity of promastigotes correlates with infectivity of *Leishmania* parasites. This correlation has been observed both among parasites from different species and as well as among isolates from the same species or clones from a single isolate [17], [18], [35]. In these studies, however, some of the strains used were isolated from infected sand flies making it impossible to correlate the ecto-nucleotidase activity with clinical features of the infected host. Thus, promastigotes from the 23 *L. (V.) braziliensis* isolates were compared in their ability to hydrolyze adenine nucleotides. Figure 2 shows that promastigotes from these isolates present a large variation in the hydrolysis of ATP, ADP and AMP. Statistical analysis of the ecto-nucleotidase activity showed that some isolates were capable of hydrolyzing significantly more nucleotides than others. In Figure 2 the results are presented in the order of increasing capacity of hydrolysis of nucleotides. It is noteworthy that the promastigotes from isolates that have high activity for ATP hydrolysis also show high hydrolytic activity for ADP and AMP (Figure 2). Thus, promastigote forms of these parasites are able not only to reduce the concentration of ATP, but also to increase the extracellular Ado concentration.

The reasons for the variability in nucleotide hydrolysis are currently unknown, however, the presence of polymorphisms in the ecto-NTPDase (our unpublished data) or ecto-5′-nucleotidase genes or differences in the expression of the enzymes on the parasite surface cannot be excluded and will be evaluated in the future. In fact, differences in the activity of ecto-NTPDase and 5′-nucleotidase have been observed in clinical isolates of *Trichomonas vaginalis* [44]. In addition, mutations in the genes of ecto-NTPDases are known to interfere with the enzyme activity and substrate specificity [45]–[48].

**Figure 3:**
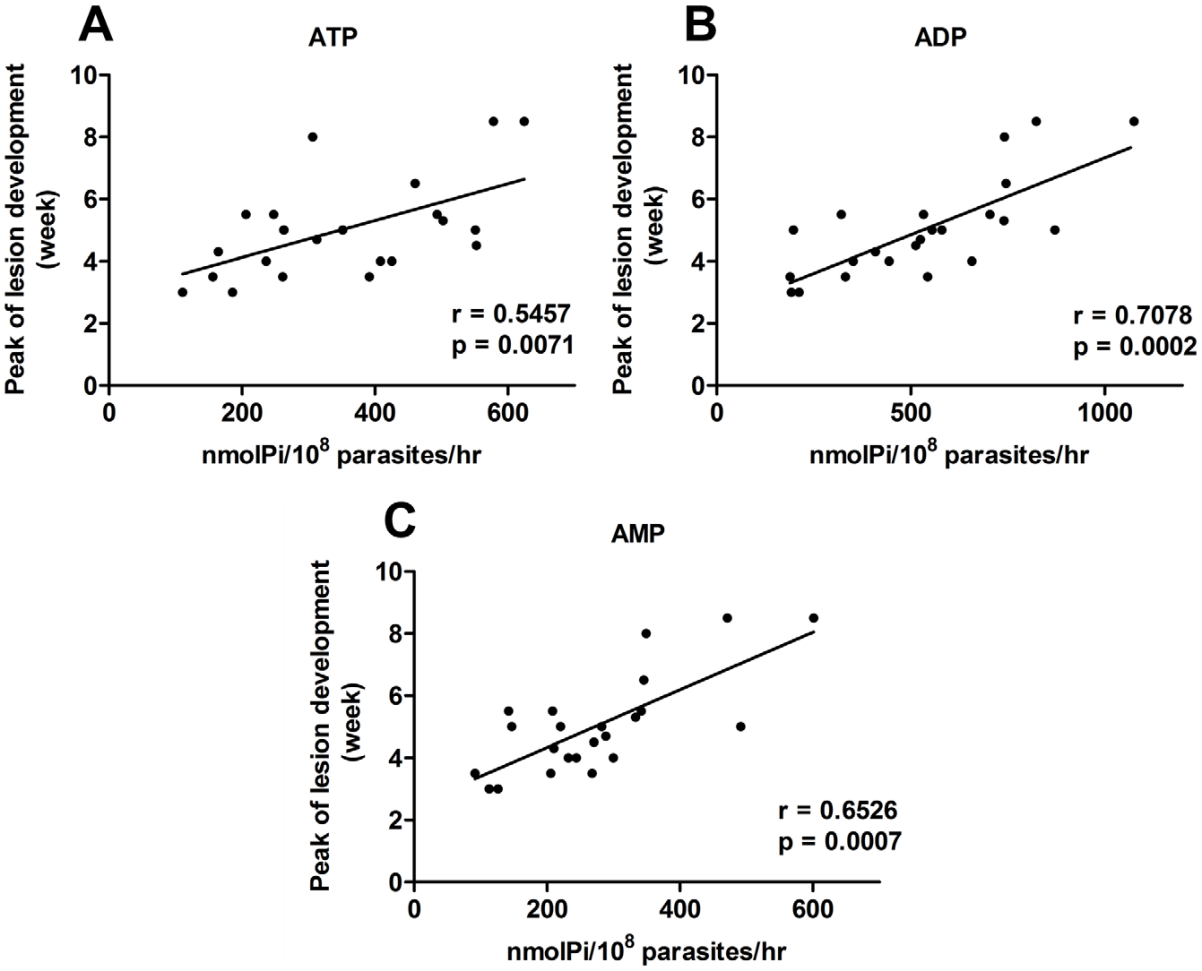
Ecto-nucleotidase activity of *Leishmania (V.) braziliensis* isolates correlates with peak injury in C57BL/6J mice. Promastigotes were isolated on the 5th day of culture and incubated with ATP (A), ADP (B) and AMP (C) for 1 hr at 30°C. Enzymatic activity was evaluated by the measurement of inorganic phosphate released. C57BL/6J mice were inoculated in the footpad of the left hind leg with 10^7^ promastigotes. Lesion sizes were measured weekly. Data-points represent the mean of the difference between the infected and uninfected contralateral footpad from two independent experiments with four mice per group with the exception of isolates RS, SAP, IMG3 and RPL5 (one experiment each). Correlation coefficient (r) calculated by the Spearman's test.

Our results do not show a correlation between the levels of ecto-nucleotidase activity and the ability of parasites to control lesion development (data not shown). However, when observing the course of infection of the different isolates we noted that some of them presented a peak of lesion development at an earlier time point than others (Figures 1A and B). Analysis of lesion development and ecto-nucleotidase activity of the promastigote forms from the 23 isolates demonstrated a positive correlation between the time necessary for the establishment of the peak of lesion development in C57BL/6J and enzymatic activity of the isolate (Figure 3). This correlation was significant for all three nucleotides analyzed. This result suggests that parasites with greater capacity to hydrolyze ATP, ADP and AMP can control the host immune response at the beginning of the infection, which would favor the multiplication of the parasite.

**Figure 4:**
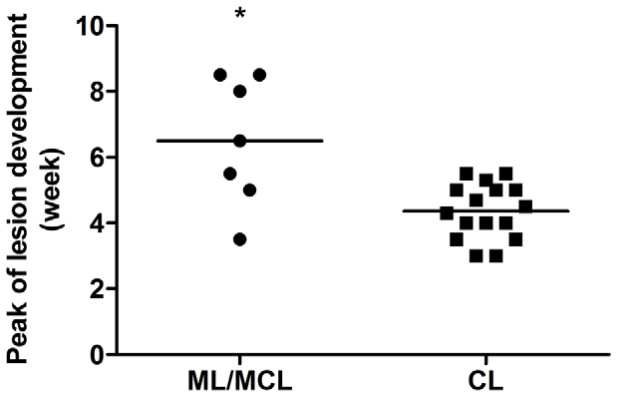
Peak of lesion development correlates with source of *Leishmania (V.) braziliensis* isolates. C57BL/6J mice were inoculated in the footpad of the left hind leg with 10^7^ promastigotes. Lesion sizes were measured weekly. The lesion size was defined as the difference between the infected and uninfected contralateral footpad from two independent experiments with four mice per group with the exception of isolates RS, SAP, IMG3 and RPL5 (one experiment each). Each point represents the time at which lesion development reached its maximum size for each isolate obtained from mucosal (ML)/mucocutaneous (MCL) lesions or cutaneous lesions (CL). The line represents the mean of the group. Statistical analysis was performed by Students's *t*-test.

Interestingly, when we segregate the isolates based on the clinical manifestation, we were able to show that isolates from mucosal/mucocutaneous lesions also showed a delayed time for peak of lesion development when compared to isolates from cutaneous lesions (Figure 4).

**Figure 5:**
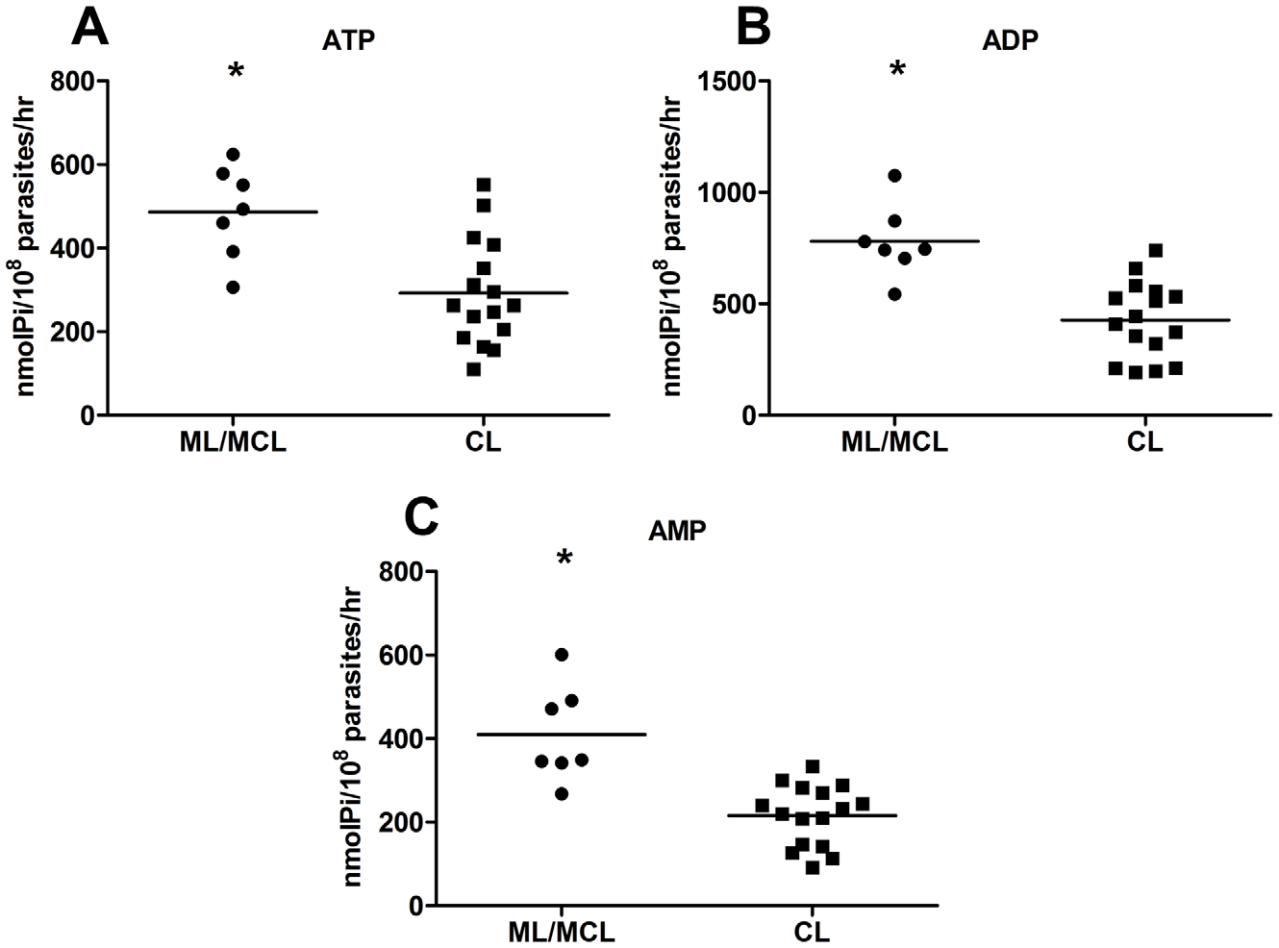
*Leishmania (V.) braziliensis* isolates obtained from patients with mucosal/mucocutaneous leishmaniasis show higher ecto-nucleotidase activity. Promastigotes were isolated on the 5th day of culture and incubated with ATP (A), ADP (B) and AMP (C) for 1 h at 30°C. Enzymatic activity was evaluated by the measurement of inorganic phosphate released. Each point represents a different isolate obtained from mucosal (ML)/mucocutaneous (MCL) lesions (A) or cutaneous lesions (CL). The line represents the mean of the group. (*) indicates statistical difference (p<0.05). Statistical analysis was performed by Students's *t*-test.

The correlation between clinical manifestation and delayed lesion development raised the question of whether promastigotes from mucosal/mucutaneous lesions showed different ecto-nucleotidase activity than those from cutaneous lesions. In agreement with the findings above, our data (Figure 5) demonstrates that promastigotes from ML/MCL presented higher ecto-nucleotidase activity than those from CL, suggesting that the delayed lesion development in infections by ML/MCL parasites is due to the increased ecto-nucleotidase activity of the promastigotes.

The delay in lesion development as a result of the increased ecto-ATPase activity of the parasite was recently demonstrated in a study using *L. (L.) amazonensis* isolates obtained from patients with different clinical forms [21]. Our results expand on this observation indicating that not only the reduction of extracellular ATP, but also, the increase in extracellular Ado may play an important role in stalling the immune response. In fact, previous data from our group demonstrated that the addition of Ado at the moment of *L. (V.) braziliensis* inoculation in C57BL/6J induced an increased lesion development and parasitism, resulting in a delay in the control of the infection [18].

The combined action of the promastigote's ecto-nucleotidases may contribute to the delay in the immune response both by decreasing the extracellular ATP concentration as well as increasing the levels of extracellular Ado. It has been demonstrated that the hydrolysis of extracellular ATP and ADP by ecto-NTPDases present in the host cells reduces the concentration of these nucleotides decreasing the activation of P2 receptors which are important stimulators of the immune system. In addition, the subsequent increase in the concentration of Ado may increase the stimulation of P1 receptors, especially the A2A and A2B receptors, which generates an immunosuppressive response [25], [49], [50]. Thus, reduction of extracellular ATP with subsequent increase in Ado levels by the promastigote's enzymes may facilitate the persistence of *Leishmania* in the host, allowing their multiplication and dissemination to other sites of body, favoring the establishment of infection. In support for this hypothesis, a recent study demonstrated that a decrease in type 1 immune response in patients with disseminated leishmaniasis may account for parasite dissemination due to decreased control of parasite growth [51]. In addition to these ecto-nucleotidases, *Leishmania* parasites also express a bifunctional enzyme called 3′-nucleotidase/nuclease that may play a significant role in the generation of Ado that may contribute to regulation of the host immune response [52], [53].

**Figure 6:**
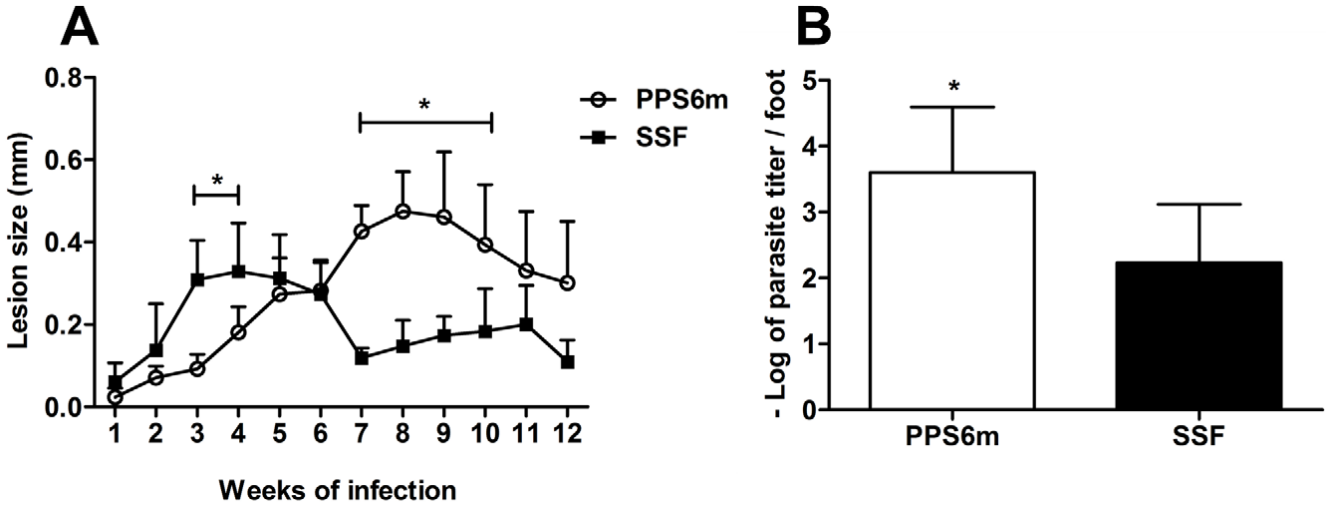
Isolate with high ecto-nucleotidase activity shows delay in lesion development in C57BL/6J mice. C57BL/6J mice were inoculated in the footpad of the left hind leg with 10^7^ promastigotes (A). Lesion sizes were measured weekly. Data-points represent the mean+SD of the difference between the infected and uninfected contralateral footpad from two independent experiments with four mice per group. (B) Parasite load in lesions. Twelve weeks after infection, animals were sacrificed and the parasite load determined by limiting dilution technique in the infected footpad. Columns represent the mean+SD of the log parasite titer from the lesions of four mice per group from two independents experiments. (*) indicates statistical difference between isolates. Statistical analysis was performed by two-way ANOVA followed by Bonferroni post-test (A) and Students's *t*-test (B).

In order to further investigate the mechanisms underlying the delayed lesion development by parasites with higher ecto-nucleotidase activity, two isolates (PPS6m and SSF) were chosen for subsequent studies of infectivity and immune response in the murine model. As previous studies raised the idea that ecto-nucleotidase activity could be related to virulence in *Leishmania* [17], [18], [35], [41], [54] as well as *Toxoplasma gondii* [55], *Trypanosoma cruzi* [42], [56] and *Entamoeba histolytica* [57], we inoculated C57BL/6J mice with promastigotes PPS6m and SSF isolates and followed lesion development during 12 weeks. As shown in Figure 6, mice inoculated with PPS6m developed a peak of lesion between the seventh and ninth weeks of infection while mice inoculated with SSF presented their maximum lesion sizes between the third and fifth weeks of infection. In addition, the lesions caused by PPS6m presented a larger parasite load than lesions caused by the SSF isolate at 12 weeks of infection.

It has been demonstrated that, in the murine model of cutaneous leishmaniasis, lesion development, rather than being a direct measure of parasite proliferation, is more related to cell migration and development of an immune response [58]–[60]. Given the differences in lesion development between PPS6m- and SSF-infected mice, we evaluated the levels of parasite proliferation and cellular infiltration at 4 and 8 weeks of infection by histological and immunohistochemical evaluation.

**Figure 7:**
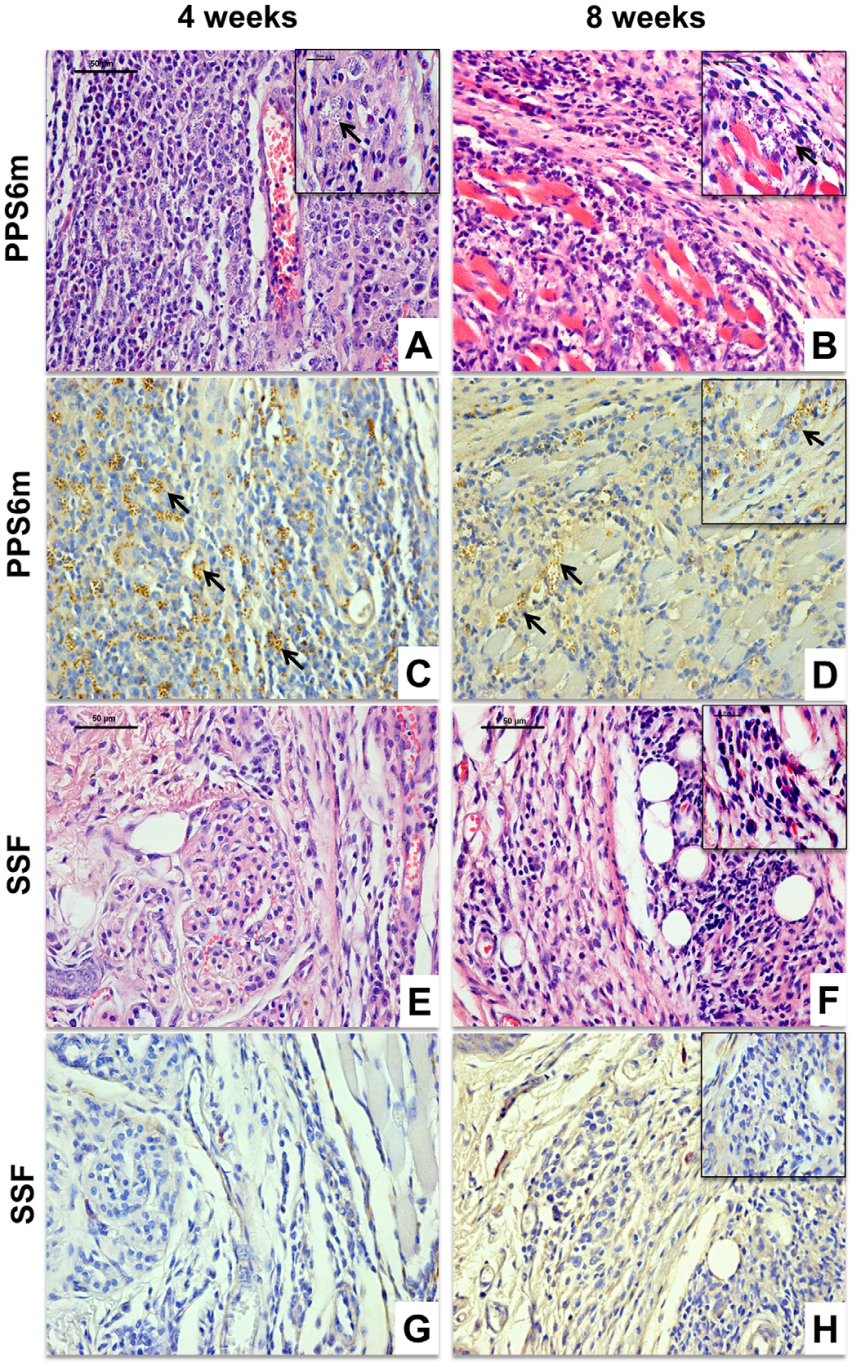
Histological (4 µm, HE) and immunohistochemical evaluation of C57BL/6J mice infected by *L. (V.) braziliensis* isolates. C57BL/6J mice were inoculated in the footpad of the left hind leg with 10^7^ promastigotes from PPS6m (A–D) and SSF (E–H) isolates for 4 (A, C, E and G) and 8 (B, D, F and H) weeks. Bar = 50 µm, 40×. Insert: Bar = 25 µm, 100×. Arrows indicate the presence of amastigotes. Figures are representative of the Histological (A, B, E and F) and immunohistochemical evaluation (C, D, G and H) of at least two independent experiments with four animals per group.

Our results show that, at four weeks of infection, mice inoculated with the PPS6m isolate showed fewer lymphocytes at the site of infection than mice infected with SSF isolate (Figures 7A and E). In addition, at four weeks of infection, parasitism was higher in mice inoculated with PPS6m than in animals infected by SSF (Figures 7C and G). Consistent with a delayed establishment of an immune response, at 8 weeks of infection, mice inoculated with PPS6m presented an intense lymphocytic infiltrate (Figure 7B) which was still associated with elevated parasitism (Figure 7D). At this time point, lesions from SSF inoculated mice, although still presenting a lymphocytic infiltrate, demonstrated evidence of tissue remodeling with very low parasitism (Figures 7F and H). These results corroborate our hypothesis that the level of ecto-nucleotidase activity present in the promastigote modulates the immune response of the host, causing a delay in the migration of lymphocytes to the lesion site.

**Figure 8:**
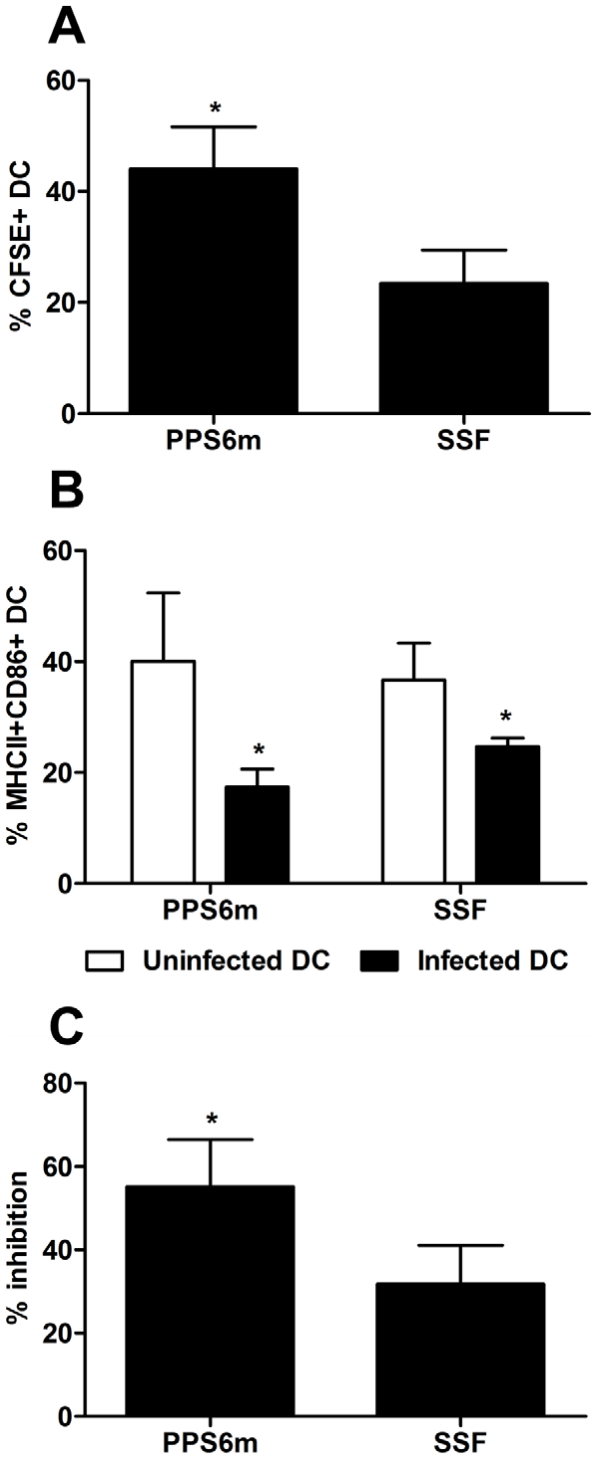
DC infection and inhibition of activation is increased in PPS6m isolate. BMDC obtained after 9 days of culture with GM-CSF were infected with CFSE-labeled metacyclic promastigotes (3 parasites/cell). After 3 hr, the cells were stimulated with 2 µg/mL LPS and then incubated for up to 17 hr. Finally, DC were analyzed by flow cytometry. DC were gated into populations of uninfected (CFSE^−^ cells) and infected (CFSE^+^ cells) BMDC, and the surface markers MHCII and CD86 analyzed in both populations. (A) Percentage of CFSE^+^ DC. (B) Percentage of MHCII^+^CD86^+^ DC. (C) Reduction of MHCII^+^CD86^+^ cells in the population of infected DC compared to uninfected DC. Bars represent the mean+SD of three independent experiments. (*) indicates statistical difference (p<0.05). Statistical analysis was performed by Students's *t*-test.

In view of the critical role DC play in orchestrating the innate and adaptive components of the immune system, we decided to investigate whether the differences in the establishment of the immune response could be attributed to differences in the ability of each isolate to interfere with DC activation and expression of co-stimulatory molecules. To this aim, we infected BMDC with CFSE-labeled metacyclic promastigotes from both isolates and evaluated infectivity and the expression of activation markers after LPS treatment (Figure 8). Our results show that PPS6 infected twice as many BMDC than SSF. In addition, while both isolates decreased DC activation, inhibition of MHCII and CD86 expression by PPS6m was significantly higher than by SSF.

It has been shown that infection of DC by *Leishmania* parasites is associated with the inhibition of cell activation [61]–[63]. Our results corroborate these findings by showing that *L. (V.) braziliensis*-infected DC are refractory to further activation by LPS, since the expression of MHCII and CD86 was decreased when compared with uninfected cells. IL-10 has been suggested as a possible factor associated with DC inhibition [64], [65]. However, no differences in IL-10 production were detected in macrophages infected by the two isolates (data not shown). Extracellular nucleotides and nucleosides have been shown to affect DC activation and migration. For example, extracellular ATP induces DC maturation and priming of Th1 cells while Ado has been shown to inhibit DC activation and pro-inflammatory cytokine production [66]–[68]. Activation of A2B Ado receptors has been shown to impair DC maturation and, consequently, decrease their ability to activate other immune cells [25]. Our results show that the isolate with high ecto-nucleotidase activity (PPS6m) presents greater capacity to inhibit the activation of BMDC, showing a possible correlation between the decreased expression of activation markers on infected DC and extracellular Ado production by ecto-nucleotidases present in *L. (V.) braziliensis* promastigotes.

**Figure 9:**
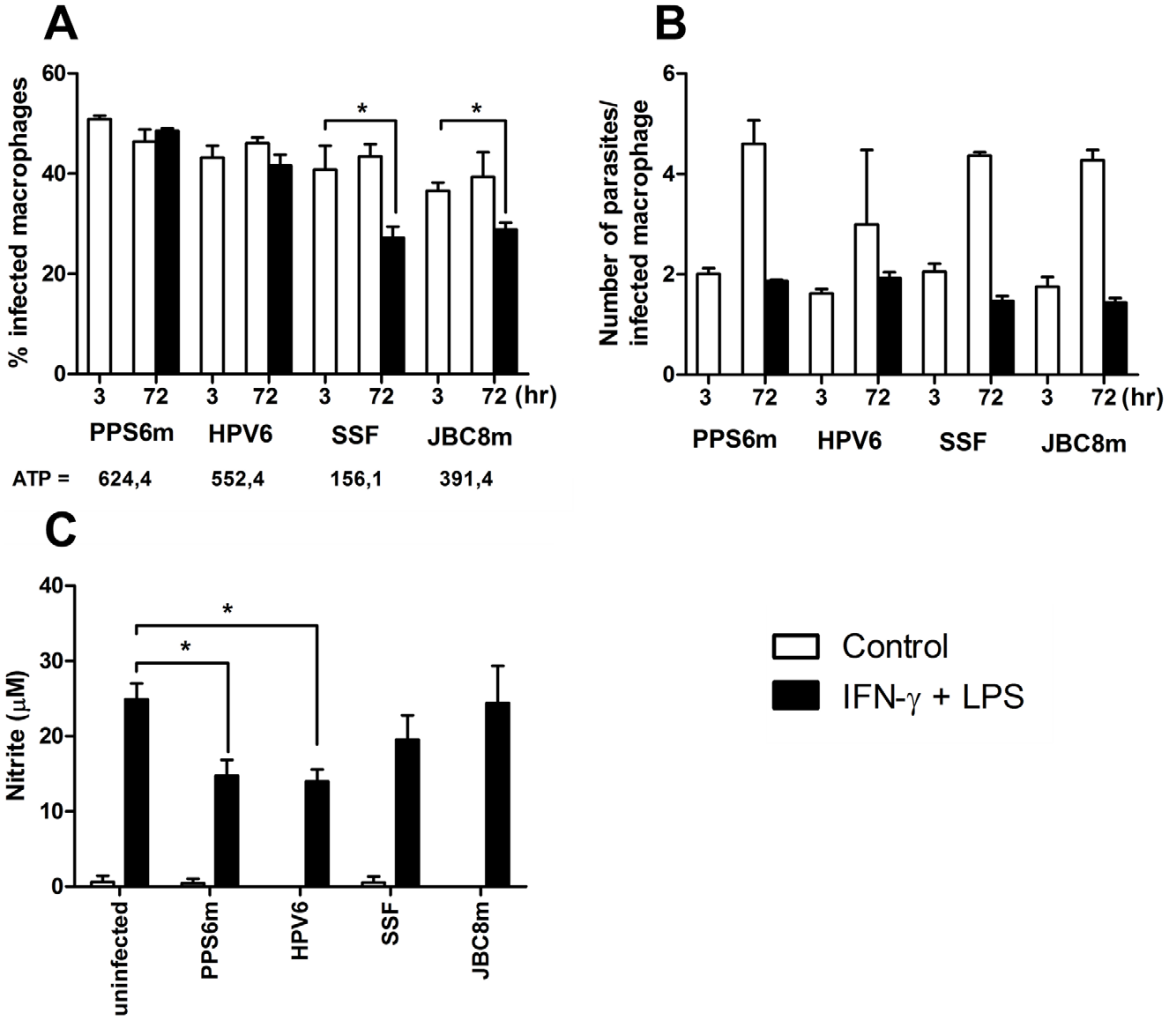
Promastigotes with high ecto-nucleotidase activity inhibit NO production by activated macrophages. J774-macrophages were infected with promastigotes of *L. (V.) braziliensis* isolates (5 parasites/cell) for 3 and 72 hr, in presence or not of IFN-γ/LPS. (A) Percentage of infected cells and ATPase activity of promastigotes (nmolPi/10^8^ parasites/hr). (B) Number of parasites per infected macrophage. (C) NO production in 72 hr supernatants. Bars represent the mean+SD of two independent experiments performed in duplicates. (*) indicates statistical difference (p<0.05). Statistical analysis was performed by Students's *t*-test.

Macrophages play a double role during *Leishmania* infection. At the same time they are responsible for parasite clearance, they also harbor the parasite allowing its multiplication in the susceptible host. To further characterize the interactions between the parasites and the host, we evaluated the *in vitro* infectivity of isolates with different ecto-nucleotidase activity in J774-macrophages (Figure 9). No differences were observed between the isolates PPS6m and SSF with regard to their ability to proliferate in J774 cells in the absence of activation by IFN-γ plus LPS. Macrophage activation prevented multiplication of parasite from both isolates (Figure 9B), however, the percentage of macrophages infected by the SSF isolate after 72 hr after activation by IFN-γ plus LPS was significantly reduced (Figure 9A) indicating that some cells were able to completely eliminate their parasites. The reasons for this ability of some cells to completely eliminate infection while other cells remain infected are unclear. Importantly, the reduction in the percentage of infected macrophages after activation was not observed in PPS6m infected cells. No differences in IL-10 production by cells infected with both isolates were observed (data not shown). However, while activation of macrophages led to a decreased production of nitric oxide in PPS6m infected cells, this was not observed in SSF infected macrophages. Taken together, these results suggest that PPS6m inhibits macrophage activation, even in the presence of IFN-γ plus LPS, which leads to increased parasite survival although with limited capacity to multiply within these cells. On the other hand, macrophage activation in SSF-infected cells seems to be more efficient thus leading to decreased percentage of infected cells.

PPS6m and SSF isolates, in addition to present high and low ecto-nucleotidase activity, were isolated, respectively, from mucosal and cutaneous lesions. In order to verify whether the observed effects on parasite survival and NO production were related to the clinical manifestation of the isolate, we performed the same experiments with two other isolates. Isolate HPV6 (obtained from cutaneous lesion) presents high ecto-nucleotidase activity while isolate JBC8m (from mucosal lesion) presents the lower ectonucleotidase activity amongst the mucosal/mucocutaneous isolates. Analysis of Figures 9A to 9C shows that HPV6 presents similar behaviour to PPS6m despite being isolated from cutaneous lesions. On the other hand, JBC8m was susceptible to macrophage activation and did not inhibit NO production similarly to what has been demonstrated to SSF. These results confirm our hypothesis that the level of ecto-nucleotidase activity of the promastigote rather than the clinical manifestation of the isolate is responsible for the resistance to macrophage activation.

The mechanisms leading to inhibition of NO production during *Leishmania* infection are not completely clear, however, molecules present at the parasite surface such as LPG and glycoinositolphospholipids (GIPL) have been shown to interfere with the expression of inducible oxide nitric synthase (iNOS) or NO production after IFN-γ+LPS stimulation [69]. We now propose that, the ecto-nucleotidase activity of the promastigote, either directly or indirectly, via the production of Ado, is also involved in macrophage modulation by inhibiting NO production thus contributing to the establishment of infection.

Taken together, the results from macrophage and DC infection by *L. (V.) braziliensis* isolates further corroborate our hypothesis [17], [18] that lesion development and parasite multiplication within the host are associated with the level of ecto-nucleotidase activity of the promastigote. Furthermore, the observation that the PPS6m isolate is able to modulate the immune response of the host by inhibiting macrophage and DC activation confirms previous findings of our laboratory with other *Leishmania* species [17], [62] and extends these results to *L. (V.) braziliensis* isolates. The inhibition of BMDC activation could explain the delay in mounting an immune response at the beginning of the infection in C57BL/6J mice thus causing the delay in lesion development. In addition, resistance to the initial activation of the macrophage would also allow for extended parasite survival.

*Leishmania* organisms have a relatively simple life cycle, characterized by two principal stages: the flagellated mobile promastigotes living in the gut of the sandfly vector and the immobile amastigotes within phagolysosomal vesicles of the vertebrate host macrophages. Our results indicate the ecto-nucleotidase activity of the promastigote is associated with decreased immune response by the host at the establishment of infection. A limited number of studies evaluated the ectonucleotidase activity in amastigotes. It has been shown that *in vitro* derived amastigotes of *L. (L.) amazonensis* present higher levels of ATP hydrolysis than promastigotes [41] suggesting the possibility of an even more pronounced effect of Ado production on the modulation of the immune response during the chronic phase of the infection. However, no direct assessment of the role of the amastigote's enzymes on the course of infection has been performed. To determine the role of ecto-nucleotidases in the propagation of the disease, we decided to evaluate the ecto-enzymes activity of amastigotes isolated from infected J774 macrophages.

**Figure 10:**
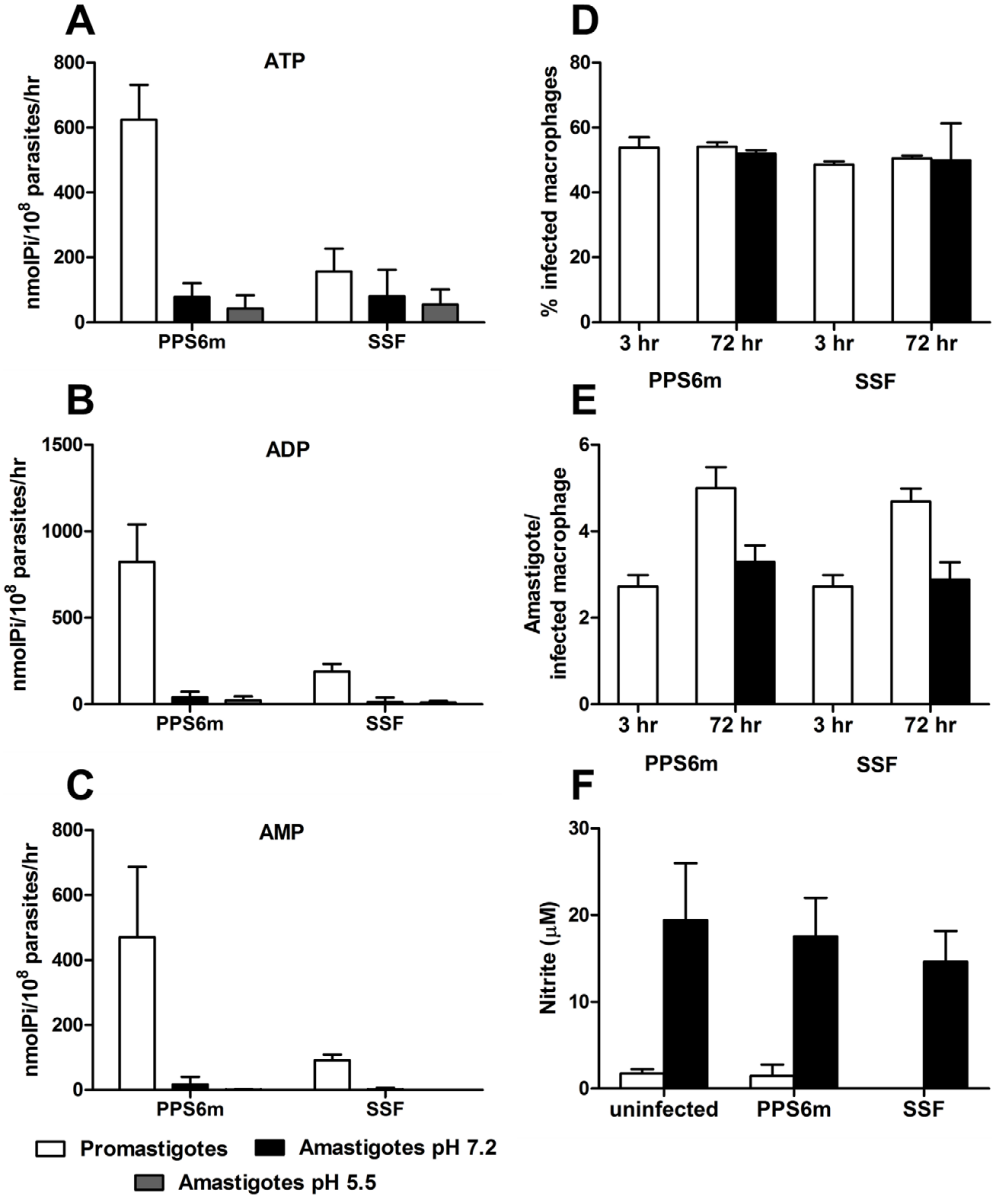
Ecto-nucleotidase activity and infectivity in J774-macrophages of amastigotes from *L. (V.) braziliensis* isolates. (A–C) Amastigotes were isolated from infected J774 macrophages and incubated with ATP (A), ADP (B) and AMP (C) for 1 hr at 30°C in pH 7.2 or pH 5.5. Enzymatic activity was evaluated by the measurement of inorganic phosphate released. Bars represent the mean+standard deviation (SD) of three or more independent experiments performed in triplicates. (D–F) J774-macrophages were infected with amastigotes of *L. (V.) braziliensis* isolates (5 parasites/cell) for 3 and 72 hr, in presence or not of IFN-γ/LPS. (D) Percentage of infected cells. (E) Number of parasites per infected macrophage. (F) NO production in 72 hr supernatants. Bars represent the mean+SD of two or more independent experiments performed in duplicates.

Contrary to what has been shown for axenic amastigotes [41], our results show that amastigotes obtained from macrophages show very low ecto-nucleotidase activity. The reason from this discrepancy is not known, but could be related to the parasite species (*L. (L.) amazonensis* versus *L. (V.) braziliensis*). Our results also show that PPS6m and SSF amastigotes do not differ in their ability to hydrolyze adenine nucleotides. Furthermore, PPS6m amastigotes presented decreased ecto-nucleotidase activity when compared to the promastigote form of this isolate. (Figures 10A–10C).

Evaluation of amastigote infectivity in J774 macrophages showed that activation of these cells by IFN-γ and LPS was able to control parasite proliferation from both isolates as previously shown for promastigotes (Figure 10E). In addition, amastigotes, which presented less ecto-nucleotidase activity than promastigotes, were not able to down modulate NO production by activated macrophages (Figure 10F). Curiously, however, this inability to reduce NO production did not correlate with a decreased percentage of infection as shown for promastigotes (Figure 10D). These results suggest that a different mechanism of parasite control might be triggered depending on whether the infection is initiated by promastigotes or amastigotes.

It has been demonstrated that the various life-cycle stages have different sensitivities to reactive oxygen species (ROS) and provoke different oxidative responses of the macrophage. Although promastigotes and amastigotes enter the macrophages by phagocytosis, the concomitant oxidative burst is substantially different. For both stages, an increase in macrophage superoxide production is seen after infection, but the response is much higher to promastigotes compared to amastigotes [2], [70]. In addition, whereas infections of macrophages by promastigote forms of *Leishmania mexicana pifanoi* induce the production of superoxide, infections by amastigotes barely induce superoxide production [71].

Nitric oxide combines with superoxide to form peroxynitrite which has been suggested to be more toxic for the amastigote than NO [2]. Also, *in vitro* studies confirmed that peroxynitrite is cytotoxic to amastigotes forms of parasites whereas nitric oxide is cytostatic [72]. On the other hand *in vitro* infections with promastigotes of *Leishmania major* show that the killing of parasite is mediated only by NO [73].

These observations support our results since NO production is inhibited in macrophages infected with promastigotes with high ecto-nucleotidase activity resulting in increased parasite survival. In infections by amastigotes, which have low ecto-nucleotidase activity, NO production is not inhibited, confirming the role of these enzymes in modulating macrophage activation. However, parasites may survive in the presence of NO, due to the incapacity of this parasite stage to induce ROS production.

Most of the prior studies on the factors that determine the development of mucosal lesions in leishmaniasis focused on the host immune response and the ability of host cells or cytokines to influence the outcome of *Leishmania* infection. In general, patients with mucosal leishmaniasis present an exacerbated inflammatory response associated with high TNF-α and IFN-γ and decreased IL-10 production [4]. In addition, decreased response to IL-10 has also been suggested as a possible cause for the intense inflammation at the site of infection [5]. Furthermore, the involvement of Th17 cells associated with the presence of neutrophils [12], high levels of pro-inflammatory monocyte chemoattractant protein (MCP-1) to recruit monocytes [10], exacerbated CD8+ activity [11] and activity of matrix metalloproteinases such as MMP-9 have been described as so important in the pathogenesis of mucosal leishmaniasis. The intrinsic capacity of MMP-9 activation of each individual might influence the intensity of macrophage efflux and dissemination of *L. (V.) braziliensis* infection to different anatomic areas [9]. Taken together, these studies indicate that patients with mucosal lesions develop a highly inflammatory response to the parasite which would seem to contradict our hypothesis. However, due to the intrinsic nature of these studies, they analyzed the patient's response after the establishment of the mucosal lesions and, therefore, cannot account for the role of the parasite in the initial phases of the infection.

The fact that different strains of the same parasite species are able to cause distinct outcomes in isogenic mice (this study and [21], [74]) indicates that parasite specific factors may also contribute to the result of the infection. According to Vendrame et al. (2010), the high arginase activity of isolates from mucosal cases suggests that this characteristic favored the development of mucosal lesions and contributed to the survival and proliferation of *Leishmania* in a hostile environment [14]. In addition, isolates obtained from patients with mucosal leishmaniasis are more resistant to NO when compared to isolates obtained from patients with cutaneous leishmaniasis [13]. More recently, it was shown that the infection of *Leishmania* by RNA virus-1 (LRV1) subverted the immune response to infection favoring promote parasite persistence and metastasis [75].

We propose high ecto-nucleotidase activity of the promastigote stage of the parasite as another parasite-related factor that could influence the clinical presentation of disease. As suggested above, high ecto-nucleotidase activity would decrease extracellular ATP and increase Ado during the initial contact of the parasite with the host at the site of infection, delaying the establishment of the response which would allow for the dissemination of the parasite. Later in the course of infection, the immune response would eventually be established and parasite control achieved. Depending on host related factors, such as decreased IL-10 receptor expression [5], mucosal destruction due to uncontrolled response would occur.

Previous results from our group suggest that early events that occur prior to amastigote transformation have an important role in the course of infection. Thus, inoculation of Ado together with *L. (V.) braziliensis* promastigotes caused an increase in lesion size and parasitism and also a delay in lesion resolution [18]. Furthermore, we also showed that the addition of Ado to *L. (L.) amazonensis* promastigotes culture medium decreases the ecto-nucleotidase activity of the parasite which correlates with decreased lesion size and parasitism [17].

Another intriguing question related to our results is the existence of isolates from cutaneous lesions with high ecto-nucleotidase activity (Figure 5). It is important to note that development of mucosal or mucocutaneous forms of leishmaniasis is generally preceded by single cutaneous lesions. It is tempting to speculate that some of the isolates from CL patients that present high ecto-nucleotidase activity would be able to induce mucosal lesions given the proper host environment. A close monitoring of these patients for decades would be needed to solve this issue.

In summary, our findings suggest that the ecto-nucleotidase activity of *L. (V.) braziliensis* isolates influences lesion development in C57BL/6J mice. The degradation of ATP and subsequent production of Ado is able to create an anti-inflammatory environment that culminates in the inhibition of the activation of DC and macrophage microbicidal mechanisms creating an environment that favors the multiplication of the parasite inside the host cell and its dissemination to other sites of the body. Although the correlation between the activity of ecto-NTPDases and parasite virulence has already been proposed, our results expand on this concept by demonstrating that Ado production may also be important and that this combination may interfere with the clinical outcome of disease. This allows us to suggest that the ecto-nucleotidases can be characterized as a virulence factor of the parasite, indicating not only a marker for the development of the mucosal/mucocutaneous clinical forms, but also a possible target for future therapeutic interventions against *Leishmania* parasites.
